# Towards Dynamic lockdown strategies controlling pandemic spread under healthcare resource budget

**DOI:** 10.1007/s41109-020-00349-0

**Published:** 2021-01-07

**Authors:** Satyaki Roy, Ronojoy Dutta, Preetam Ghosh

**Affiliations:** 1 University of North Carolina, Chapel Hill, USA; 2 Deep Run High School, Glen Allen, VA USA; 3 Virginia Commonwealth University, Richmond, USA

## Abstract

COVID-19 is one of the deadliest pandemics in modern human history that has killed nearly a million people and rapidly inundated the healthcare resources around the world. Current lockdown measures to curb infection spread are threatening to bring the world economy to a halt, necessitating dynamic lockdown policies that incorporate the healthcare resource budget of people in a zone. We conceive a dynamic pandemic lockdown strategy that employs reinforcement learning to modulate the zone mobility, while restricting the COVID-19 hospitalizations within its healthcare resource budget. We employ queueing theory to model the inflow and outflow of patients and validate the approach through extensive simulation on real demographic and epidemiological data from the boroughs of New York City. Our experiments demonstrate that this approach can not only adapt to the varying trends in contagion in a region by regulating its own lockdown level, but also manages the overheads associated with time-varying dynamic lockdown policies.

## Introduction

COVID-19 is the latest addition to the long list of pandemics that scarred human history during the last millennium (Coronavirus 2020). COVID-19 has followed a similar course like the plague, flu and Ebola and claimed nearly 1.34 million lives globally as of November 2020, while its severity continues to burgeon in the US, UK, Brazil and parts of Asia (Coronavirus 2020; Mortality 2020). In the absence of any credible vaccination treatment (Adhikari et al. 2020), social distancing and lockdown measures emerged as the modus operandi to negate the surge in infection numbers. However, the projected slump in the world economy has compelled the policymakers in the developing nations to consider easing the mobility restrictions.

The primary concern of the health officials regarding this lockdown relaxation is the increased social interaction leading to a possible spike in infected counts which the healthcare facilities of even the most developed nations may be ill-equipped to combat (Coronavirus 2020). Such fears are a throwback to April 2020 when the national media reported a shortfall in intensive care unit beds and rising fatalities in the wake of the projected wave of COVID-19 cases (Coronavirus 2020). Recently, as many as 14 US states have been compelled to reconsider their decision to ease lockdown restrictions, as the infection numbers soared and threatened to overwhelm the available healthcare resource (Weeks 2020). The present state of affairs necessitates the design of dynamic lockdown policies that incorporate the economic and epidemiological ramifications of future pandemics and, more importantly, the healthcare resource budget of a region.

### Related works

#### Susceptible-exposed-infected-death (SEIRD) epidemic model

SEIRD (see “Data” section) has been used to model the effects of immunity, demography as well as social distancing on the spread of COVID-19. Gharakhanlou applied SEIRD to create an agent-based simulation to show the effects of social contact and propose potential mitigation measures to contain the spread of COVID-19 in Urmia city, Iran (Gharakhanlou and Hooshangi 2020). Ghanam et al. present a bayesian method to estimate the parameters for the SEIRD model and quantify the impact of government intervention measures on infection spread (Ghanam et al. 2020). Lattanzio et al. studied the interrelationship of lockdown and mobility in Lombardy and London as well as the ill-effects of flouting social distancing regulations (Lattanzio and Palumbo 2020). Keeping in mind, the debate over whether the recovered individual can be reinfected, Malkov et al. utilized SEIRD to study the effects of mitigation measures on reinfection and no-reinfection scenarios (Malkov 2019). Piccolomini et al. adapted the SEIRD with time-varying transmission rates to model restrictions imposed by the government to combat COVID-19 (Loli Piccolomini and Zama 2020; Piccolomini and Zama 2020).

#### Machine learning approaches

The lack of prior knowledge on COVID-19 leaves the policymakers ill-equipped to design mitigation strategies. Epidemiologists, health experts and computer scientists have joined forces to identify the socioeconomic factors and their implications on contagion as well as economic downturn (Adhikari et al. 2020)—this includes using machine learning (ML) to build prediction models on epidemiological and clinical data. Given existing clinical data, prediction models (Wynants 2020) and therapeutic approaches can help identify vulnerable groups (Alimadadi et al. 2020; Randhawa et al. 2020). Epidemiologists are trying to identify spread dynamics of COVID-19. Holmdahl and Buckee (2020) analyzed the pros and cons of forecasting models that make predictions through curve fitting or mechanistic models, while supervised and unsupervised ML is helping trace the trends in infection dynamics (Wang et al. 2020). Khan et al. used regression tree analysis, cluster analysis and principal component analysis on Worldometer infection count data to gauge the variability and effect of testing in the prediction of confirmed cases (Khan et al. 2020). Also, Roy et al. performed regression analysis to identify pre-lockdown factors that affect the post-lockdown pandemic numbers (Roy and Ghosh 2020).

### Issues in vaccine production and supply

There is a mistrust brewing over the efficacy of the vaccines. The public at large is sceptical about the “rush” to put out the vaccine before adequate bouts of clinical testing (Mistrust 2020). Many believe that the undue optimism in releasing the vaccine can have adverse health ramifications. Moreover, governments continue to plan to expedite this process by parallelizing the steps of research and trials as well as industrial-scale manufacture of vaccines (Testimony 2020). Third, vaccines must be affordable and accessible by all irrespective of the social or economic strata they belong to. This poses a policymaking problem to guarantee the equity of resource allocation. There are several aspects to vaccine allocation from the standpoint of policymaking per se. There is an economic angle associated with the distribution of vaccines. Given that the vaccines will be stored at warehouses, it becomes crucial to minimize the economic overhead of transporting vaccines to the affected zones. Finally, there are political and market forces that may obtrude the vaccines from reaching the worst-hit states, making fairness a key factor for regulations.

Other epidemiological factors, such as population density, number of susceptible individuals and the infected ratio, play a role in the dynamics of infection spread (Farman et al. 2018). Population density governs the “contact with susceptible individuals” resulting in contagion (Tarwater and Martin 2001; Rocklöv and Sjödin 2020). Also, the spread and sustenance of an epidemic is contingent on whether there is an adequate number of susceptible hosts in the total population (Principles 2020). Finally, since the spread of infection depends on contact between a susceptible host and infected individual (Korolev 2020), high percentage of infected people in the total population (i.e., infected ratio) leads to contacts contributing to the spread of the outbreak. Thus, the policymaker also needs to factor in some or all of these aspects in determining the vaccine distribution policies across regions. Most importantly, in absence of adequate clinical trials, the allocation strategies must take into consideration the innate uncertainty in the extent of immunity such vaccination can achieve (Lurie et al. 2020).

*Contributions.* In this work, we conceive a dynamic pandemic lockdown strategy that factors in public health infrastructure of a geographical region. The proposed approach built upon reinforcement learning (RL) allows agents to take decisions to maximize reward, while adapting to a complex and uncertain environment (Tuyls and Weiss 2012; Pecka and Svoboda 2014). We create an agent-based simulation environment running the ordinary differential equation-based SEIRD epidemic model (Hethcote 2000) (discussed in “Scenario” section). A geographic region, modeled as an agent, is classified into zones, and each zone has a healthcare budget commensurate with its gross domestic product (GDP). Each agent (or zone) periodically invokes the RL model to select a discrete lockdown level based on two different models (1) *average velocity* of the individuals in that zone and (2) *contact index*: a measure of the average contact of individuals within a borough independent of the demographic factors like population density, both of which affect the rate of human contact. Both these models showcase the generalizability of our proposed framework which can readily be extended to other factors that may affect the contact rates of infected individuals with the susceptible population. We employ the queueing model to ensure that the number of hospitalizations is constrained by its available healthcare resource budget.

We design a simulation environment using the Python Simpy library (Matloff 2008) that operates on the real demographic and epidemiological data from the 5 boroughs of New York City, namely Manhattan, Bronx, Brooklyn, Queens and Staten Island. We introduce a realistic model that employs real mobility traces and epidemic status of individuals in each zone to determine inter-zone mobility. Moreover, we formulate a KL divergence minimization problem to learn the epidemiological parameters that yield the daily infected curve. The healthcare budget of each borough comprises a quanta of hospital facilities, each modeled, as per single server queueing theory, as a server with an inflow and outflow of patients. Our experiments show that despite the heterogeneity in infection dynamics, each borough effectively modulates its mobility to curb infection spread and consequent hospitalization. We also demonstrate how several simulation parameters can help regulate the overall cost associated with the time-varying dynamic lockdown strategy.

## Materials and methods

Let us discuss the data used and the components of the proposed approach.

### Data

- We consider 5 towns, called *boroughs*, in New York City (NYC), viz. Bronx, Brooklyn, Manhattan, Queens and Staten Island. We obtain the borough data, such as Gross Domestic Product (GDP), population density, etc., from Wikipedia (Neighborhoods 2020). COVID infection and deaths are taken from The City (Coronavirus 2020) based on records of Department of Health and Mental Hygiene. We use NYC Health records (Nyc health 2020) that show daily infected from March-August 2020 from New York Department of Health.
- We source the mobility data of NYC traffic from NYCOpenData (Nycopendata 2020)—a repository for fields ranging from city government, education, environment, health to public safety, recreation, social services and transportation. The stated data (spanning a period from 2014 to 2019), collected by the Department of Transportation of New York Metropolitan Transportation Council (NYMTC), has the following fields: *ID*, *road name*, *source and destination intersecting street name*, *compass direction*, *date* and *time*. We calculate the transition matrix (see “Inter-zone mobility model” section) that captures the probability of travelling within and across boroughs.

**Fig. 1:**
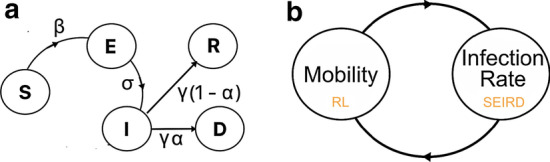
System model: **a** state transitions in the susceptible, exposed, infected, recovered, death (SEIRD) model with the associated parameters; **b** a closed loop of reinforcement learning and SEIRD that controls human contact and infection of a zone

### SEIRD epidemic model

We adapt the susceptible-exposed-infected-recovered-death (SEIRD) model (see Fig. 1a) (Hethcote 2000). The *susceptible* (S) class comprises individuals who are not exposed to the infection. Once exposed to infected individuals, they may transfer to the *exposed* (E) category, and this transition is controlled by a value $\beta$ (that is not necessarily a probability). The E class are asymptomatic or untested individuals, who transition to the (tested) *infected* (I) with probability $\sigma$. The individuals in *I* transition to another state with a probability $\gamma$, either *recovered* (R) or *dying* ($\mathcal {D}$) with probabilities $1 - \alpha$ and $\alpha$.

### Ordinary differential equations

Ordinary Differential Equations (ODE) model estimates the state transitions $S \rightarrow E \rightarrow I \rightarrow R \rightarrow D$ by solving the following set of ordinary differential equations (Korolev 2020). We implement the ODE model on Python Odeint library (Ahnert and Mulansky 2011).

1 $$\begin{aligned}&\frac{dS(t)}{dt} = - \frac{\beta S(t) I(t)}{N} \end{aligned}$$

2 $$\begin{aligned}&\frac{dE(t)}{dt} = \frac{\beta S(t) I(t)}{N} - \sigma E(t) \end{aligned}$$

3 $$\begin{aligned}&\frac{dI(t)}{dt} = \sigma E(t) - \gamma I(t) \end{aligned}$$

4 $$\begin{aligned}&\frac{dR(t)}{dt} = (1 - \alpha ) \gamma I(t) \end{aligned}$$

5 $$\begin{aligned}&\frac{dD(t)}{dt} = \alpha \gamma I(t) \end{aligned}$$

Here $\beta$ is the contact rate, *N* is the total population, $\sigma$ is the incubation period, $\gamma$ is the duration of infection and $\alpha$ is the fatality rate. In Eq. 1, $\beta = p \times C$, where *p* is the infection probability and *C* is the individual contact rate. Since, contact rate *C* can vary for different zones, we represent it as a product of a constant terms (density $\rho$) and a variable term (*contact index* *k*), i.e. $C = \rho \times k$. We assume that the social contact and interaction among individuals at a zone follows the collision among ideal gas molecules in a homogeneous mixture. We can also calculate $C = \sqrt{2} \pi d^2 \rho v$, where *d* is the collision diameter, $\pi d^2$ is the cross-sectional area, $\rho$ is the population density and *v* is the mean velocity (Hu et al. 2013). The COVID-19 specific epidemic parameters for ODE used in our experiments are discussed at the beginning of “Results” section.

### Scenario

We create an agent-based simulation environment using the Python Simpy library (Matloff 2008), where each zone (termed *borough*) is an agent with a predefined initial population of susceptible, exposed, infected, recovered and dead individuals. Each zone also has an initial lockdown level $l_i$ (where $1 \le i \le \chi$); the higher the lockdown level, the lower is the mobility (measured in terms of the average velocity *v* of individuals) in a zone. Specifically, the assumption is that the average velocity of individuals at a particular zone is proportional to their distance covered, which in turn correlates with higher overall social contact, mixing and contagion. The new infected count *I* is determined by periodically (i.e., after interval $\eta$) invoking the ODE SEIRD model (discussed in “Ordinary differential equations” section). Since higher mobility leads to greater contact and contagion, each zone invokes the reinforcement Q-learning module (see “Reward function” section) to learn and determine an updated *v*. The objective is to maximize mobility, while ensuring that the number of hospitalizations is within the healthcare resource budget of the zone. We assume that the healthcare resource budget of borough *b* (is commensurate with the number of hospital beds and) is measured in terms of its overall GDP using the equation below:

6 $$\begin{aligned} |H_{b_i}| = \frac{GDP_{b_i} - \mu (GDP)}{\sum _{b_j \in B}GDP_{b_j}} \times bG \end{aligned}$$

Here $GDP_{b_i}$ is the GDP of borough $b_i$ and *bG* is the baseline hospital bed count. This formulation ensures that the number of beds allotted to each borough is proportional with its GDP. Finally, we measure the number of hospitalizations as a fraction, say *k*, of the newly infected population. Finally, Fig. 1b shows that the proposed system is a closed loop of reinforcement learning and SEIRD models controlling the mobility and infection of a zone.

### Inter-zone mobility model

Given a region with a set of geographical sub-regions (or zones) *B*, the frequency matrix $F \in \mathbf {M}_{|B| \times |B|} (\mathbb {R})$ is created from the human mobility traces, where $f_{i, j} \in F$ denotes the number of trips made from zone $b_j \in B$ to $b_i \in B$. We generate a transition matrix $A \in \mathbf {M}_{|B| \times |B|}$ performing column normalization of *F*. Each element of the matrix $a_{i, j} \in A$ is the probability of making a trip from $b_j$ to $b_i$. The frequency (and transition matrix) captures the overall mobility trends within and across zones of any given region. A preassigned number of people migrate from one region to another based on the following inter-zone mobility procedure employing the transition matrix *A*.

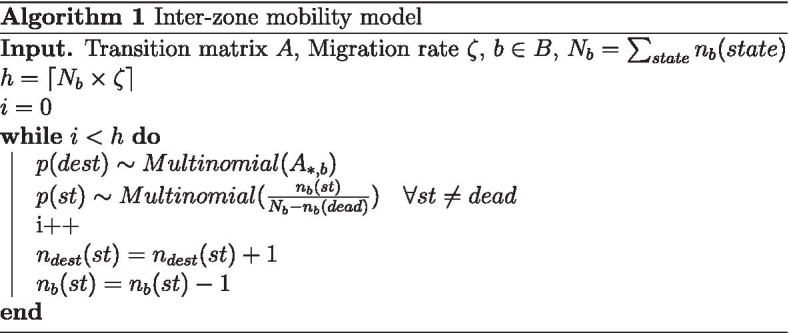



**Description.** Procedure 1 is invoked periodically by each borough *b* of population $N_b$, where $h = \lceil \zeta * N_b \rceil$ people move, where migration rate $\zeta$ ranges between 0 and 1. For a moving person, the destination borough $dest \in B$ is chosen based on a multinomial distribution on the b-th column of transition matrix *A*, i.e., $A_{*,b}$. Similarly, state of the alive person *st* is proportional to the fraction of people within that state, i.e. $\frac{n_b(st)}{N_b - n_b(dead)}$. Following this, the algorithm decrements the number of people in *b* with state *st*, $n_b(st)$, and increments $n_{dest}(st)$ to reflect migration from borough *b* to *dest*.

### Minimization of Kullback–Leibler divergence

The Kullback–Leibler (KL) divergence measures the difference between one probability distribution from another reference probability distribution (Kullback and Leibler 1951). Given two probability distributions *P* and *Q*, it is measured as:

7 $$\begin{aligned} D_{KL} (P || Q) = \sum _{i = 1}^n P(x_i) \times (\log P(x_i) - \log Q(x_i)) \end{aligned}$$

Given *N*, $\sigma$, $\alpha$, *p*, $\rho$ and $\gamma$, we learn the two parameters *v* and $E(t = 0)$ to generate a reference curve that fits actual infected curve $I_f$ having the least KL divergence from the actual (or reference) daily infected curve for a given region $I_a$. The fitting optimization problem is formulated as:

8 $$\begin{aligned} \min \limits _{v, E(t = 0)} D_{KL} (I_a || I_f (N, \sigma , \alpha , p, \rho , \gamma )) \end{aligned}$$

### Modeling hospitalization queue

A borough $b \in B$ has a healthcare capacity $H_b$. We model a hospitalization facility $h \in H_b$ as a single server with patient arrival rate *a* and treatment (or service) rate *r*. As per single-server queueing model, a waiting line or a *queue* is formed when a server has more than 1 person in the system. Let $p^h(i)$ be the probability that there are *i* people in the system of server *h*; then probability of a queue forming in front of facility *h* is given by $p^h_{queue}= 1 - p^h_0 - p^h_1$. We assume that the *arrival-to-service* ratio $\delta = \min (1, \frac{a}{r})$, making $p^h_0 = 1 - \delta$ and $p^h_1 = p^h_0 \times \delta$ as per single-server queueing model. At any given time *t*, we estimate the mean probability of queue as $p_{queue} = \frac{\sum _h p^h_{queue}}{|H_b|}$. Once hospitalized, a patient may transitions to dead (D) or recovered (R) categories with *hospital fatality rates* $\alpha _h$ and $1 - \alpha _h$, respectively.

### Reinforcement Q-learning model (RL)

Q-learning (Watkins and Dayan 1992), invoked every *W* hours, allows agents (i.e., zones) to take decisions to maximize reward while adapting to an uncertain environment. Given a set of possible actions $A = \{ a_1, a_2, \cdots , \}$, each agent maintains a Q-table that records the past rewards the agent has received for an action. Thus, the Q-table, *Q*, is a matrix $\mathbb {R}^{|A| \times |A|}$. We modulate the *exploration vs exploitation* factor allowing the RL model to pick a random action with a probability *ep*. Note that *ep* undergoes a decay by a factor *dc* ($e, d \in [0, 1]$) after each run of the model. Next, we discuss the action space and reward.

#### Action space

The rows and columns of *Q* represent current action and next action, respectively. In addition to the $\chi$ lockdown levels $l_1, l_2, \cdots , l_{\chi }$ (explained in “Scenario” section), the probability of queue $p_{queue}$ is discretized into $\omega$ levels $w_1, w_2, \cdots , w_{\omega }$ ranging from low to high. This makes the search space *A* a set of combinations of lockdown levels and $p_{queue}$ levels, i.e., $A = \{ (l_1, w_1), (l_1, w_2), \cdots , (l_\chi , w_\omega )\}$. It is noteworthy that the RL model of each zone can only control the lockdown level *l* (i.e., velocity). Consequently, a zone can transition from current action $a = (l_y, w_j)$ to another state $a = (l_z, w_j)$, where $1 \le y, z \le \chi$, and the new $p_{queue}$ will be determined by the number of new infections spawned by the change in lockdown level.

#### Reward function

Recall from our discussion in “Ordinary differential equations” section, the rate of human contact is controlled by the contact rate $C = \sqrt{2} \pi d^2 \rho v = \rho k$. Since *C* is a function of both average velocity *v* and contact index *k*, we devise the two reward function that incentivizes conflicting goals of (1) high human contact based on either *C* or *k* and (2) low hospital occupancy (i.e., low $p_{queue}$). It is calculated as:

9 $$\begin{aligned} R = \frac{v}{\max (\mathbf {V})} \times e^{- p_{queue}} \text {or} \frac{k}{\max (\mathbf {K})} \times e^{- p_{queue}} \end{aligned}$$

Here, the first term is the permitted velocity $v \in V$ (or contact index $k \in K$) of a borough normalized by the maximum velocity $\max (\mathbf {V})$ (or contact index $\max (\mathbf {K})$) and the second term penalizes high hospital occupancy of a zone. Later in Fig. 3b, we show that the $e^{- p_{queue}}$ drops with the increase in $p_{queue}$.

**Table 1:** Default parameter values

Parameter	Notation	Value
Number of iterations	–	100
Simulation duration	*T*	180 days
Number of boroughs	|*B*|	5
Number of lockdown levels	$\chi$	6
Interval for invoking ODE	$\eta$	12 hours
SEIRD parameters(Korolev 2020)	$\sigma , \gamma , \alpha$	0.25, 0.1, 0.05
Interval and initial number of new infection	$\zeta , k$	30 days, 200
Migration rate	$\zeta$	0.01
Treatment rate	*r*	0.0029
Hospital fatality rate	$\alpha _h$	0.2
SEIRD infection probability	*p*	0.01
Collision diameter	*d*	1 m
RL probability of random action	*ep*	0.75
RL decay factor	*dc*	0.99
RL transition window	*W*	5.5 days
Number of probability of queue levels	$\omega$	3
Levels in probability of queue	$w_1, w_2, w_3$	$0 - 0.33$, $0.33 - 0.66$, $0.66 - 1.0$

#### Pearson correlation coefficient

It captures the linear relationship between two variables. The values of 1 and $-1$ represent high positive and negative correlations, while 0 represents uncorrelated variables. Given two distributions *X* and *Y*, it is $\frac{cov(X,Y)}{\sigma _X \times \sigma _Y}$, where $\sigma _X$ and *cov*(*X*, *Y*) are standard deviation and covariance  (Benesty et al. 2009).

#### Overhead of lockdown

It is imperative to recognize that imposing time-varying lockdown is cost-intensive, as the news of the updated lockdown level must be disseminated among the public through electronic and print media. Moreover, since lockdown affects every aspect of human life, temporal lockdowns can have wide-ranging social and economic implications. We assume that the cost of lockdown is directly proportional to the number of transitions in lockdown levels $l_i$. This cost can be controlled by regulating two parameters: (1) *lockdown window*, $\tau$ – duration (in hours) before the RL model is invoked and $l_i$ is re-evaluated and (2) *lockdown threshold*, $\alpha$ – real value between 0 and 1, such that RL model is invoked by a borough only if $|p_{queue} (t) - p_{queue} (t - 1)| > \alpha$.

## Results

The simulation environment is implemented in Python. The demographic, epidemiological and human mobility data sources for NYC boroughs are discussed in “Data” section. We define the following 4 lockdown levels in terms of contact index: $l_1, l_2, l_3, l_4$ with contact index $k = 4.4 \times 10^{-6}, 1.7 \times 10^{-5}, 3.1 \times 10^{-5}$ and $4.4 \times 10^{-5}$, respectively. The results section has been organized into three broad headings: (1) inter-zone mobility model, (2) relationship among contact index (or velocity), healthcare capacity and reward and (3) dynamics among contact index, infection and hospital capacity in boroughs. All the parameters (and their default values) are summarized in Table 1.

### Inter-zone mobility model

**Fig. 2:**
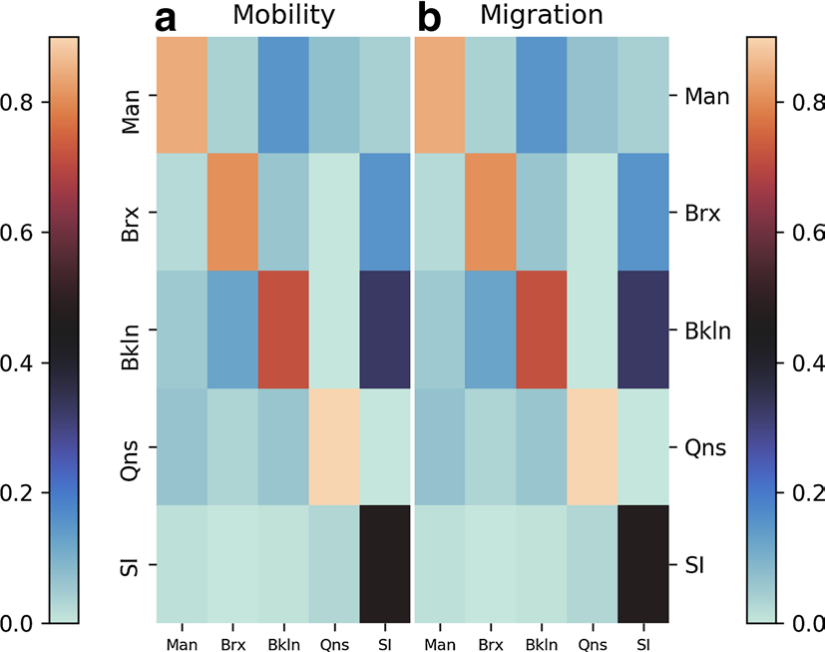
Mobility: **a** the transition (or mobility) matrix based on NYC traffic data (Nycopendata 2020), and **b** the migration matrix produced by employing the inter-zone mobility model

The migration of people across boroughs are dictated by the inter-zone mobility model (refer “Inter-zone mobility model” section) to mimic the transition matrix. We generate this migration matrix where each element (*i*, *j*) is the number of trips made from source borough *j* to *i* normalized by the column sum. Figure 2 shows the migration matrix (right) where each element has the same color (i.e., mobility probabilities) as the corresponding element from the transition matrix.

**Fig. 3:**
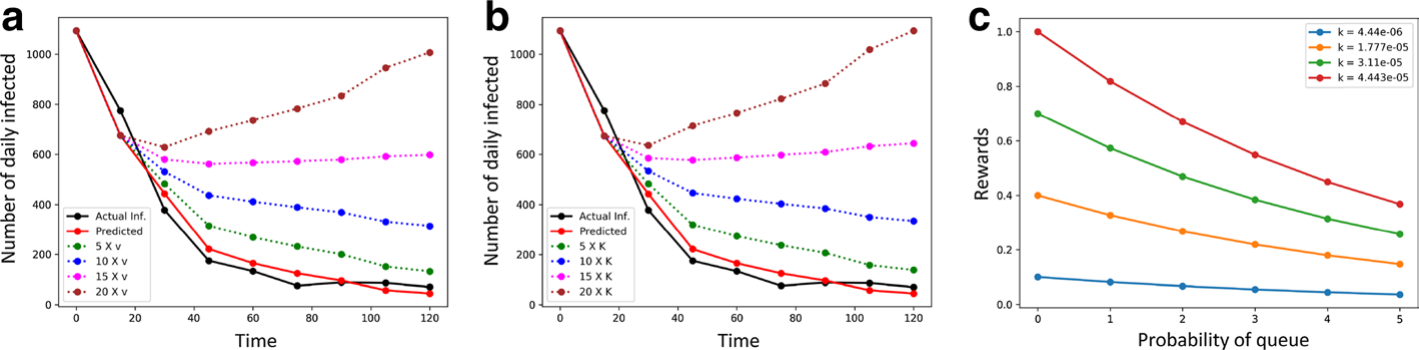
The effects of **a** average velocity *v* and **b** contact index *k* on the daily infected numbers; **c** rewards for different probability of queue ($p_{queue}$) and contact index *k*

### Relationship among contact index (or velocity), healthcare capacity and reward

We first apply KL divergence minimization (explained in “Minimization of Kullback–Leibler divergence” section) to learn three SEIRD parameters (average velocity *v*, fraction of initial exposed population $E(t = 0)$ and infection duration $\gamma$) for each borough based on COVID-19 daily confirmed cases (discussed in “Data” section). (The values of all other parameters used in the ODE model (Eq. 1 - 5) have been taken from Korolev (2020).)

Figure 3a shows the fit line (shown in solid red line) obtained by solving the optimization on the *post-lockdown* daily infected numbers (solid black line); the corresponding parameters are $v = 0.1$ km/h and $E(t = 0) = 1.82 \times 10^{-4} \times$ total population (*N*). In Fig. 3b, we apply the same fitting to obtain an equivalent contact index $k = 4.63 \times 10^{-06}$. For either case, we show the surge in the projected daily infected numbers for lower lockdown levels (i.e., higher velocities and corresponding contact index levels) shown in different colors. This shows that the proposed approach is fairly generalizable, as the roles of *k* and *v* are interchangeable in the RL reward function.

**Fig. 4:**
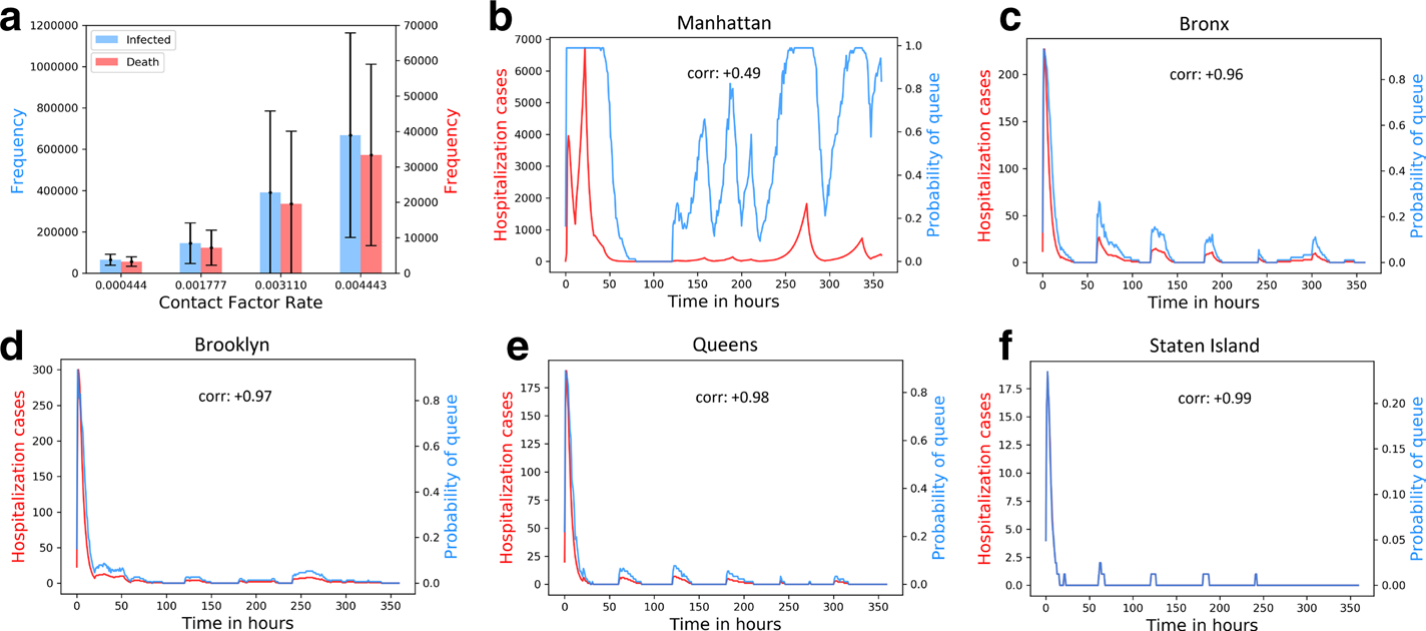
Dynamics among contact, infection and hospital capacity in NYC boroughs: **a** change in mean infected and death numbers across boroughs with contact indices *k*; **b**–**f** Change in number of hospitalization and probability of queue over time, for each borough

#### Reward function

We analyze the variation in the reinforcement Q-learning reward (Eq. 9) for different input parameter values of hospital capacity (measured in terms of probability of a hospital queue $p_{queue}$) and the lockdown levels (estimated by velocity *v* and contact index *k*) in each borough. Figure 3c shows that the reward function balances the trade-off between *k* and $p_{queue}$. Evidently, the reward is low if any of the two conditions hold true: *k* is low or $p_{queue}$ is high.

### Dynamics among velocity, infection and hospital capacity in boroughs

We study how high contact (or *k*) affects infected number, which in turn affects hospital capacity. Figure 4a shows that the mean total infected and death numbers across boroughs increase with velocity. Both the mean infected and death numbers exhibit a fairly high deviation from the mean, suggesting that there is a high variation in dynamics of infection spread across NYC boroughs. Figure 4b–f shows the time-varying number of hospitalizations for each borough is understandably correlated with the probability of queue $p_{queue}$.

**Fig. 5:**
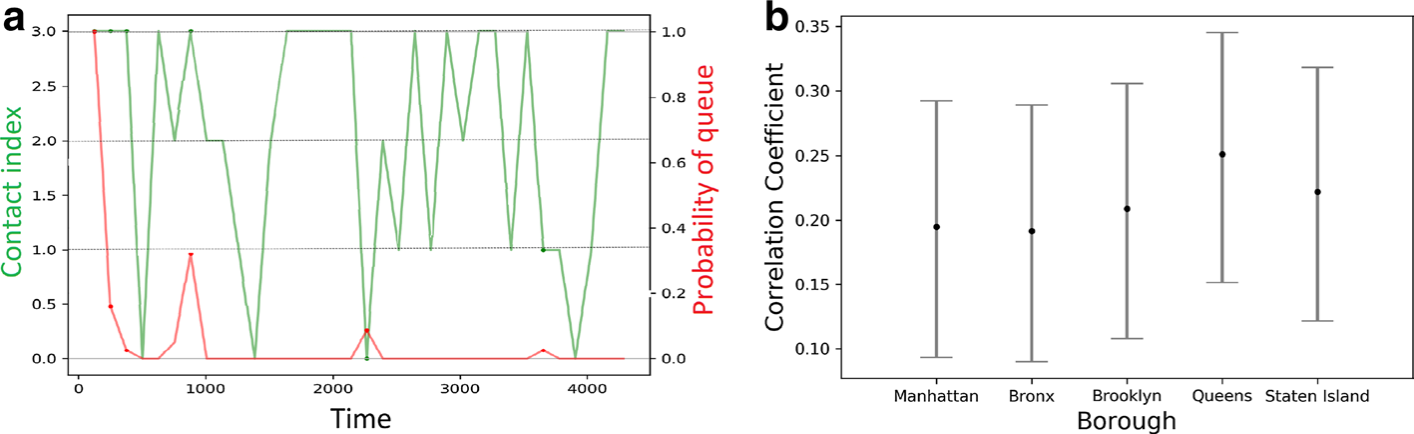
Adaptive mobility with Q-learning: **a** Variation in contact index with $p_{queue}$; **b** range of mean Pearson correlation coefficient (with $95 \%$ confidence) for the 5 boroughs

#### Adaptive mobility with reinforcement Q-learning

We study how boroughs invoke the RL model (discussed in “Reward function” section) to adapt its contact index (i.e., *k*) with changing hospital queue $p_{queue}$. Given 4 equi-spaced contact index levels *k* ranging between $4.4 \times 10^{-6}$ to $4.4 \times 10^{-5}$. Figure 5a shows the overall change in *k* with the dynamics of $p_{queue}$. The phase changes in $p_{queue}$ and the corresponding phase changes in *k* are denoted by red and green curves, respectively. Evidently, the RL model is able to adapt mobility to keep the infection counts (and the associated hospitalizations) under check (with mean absolute difference between the levels of *k* ($l_k$) and $p_{queue}$ on a scale of 0 to 1 $\sum _t |0.33 \times l_k(t) - p_{queue}(t)| = 0.63$. Next, we record the mean correlation between 4 levels of *k* and $- p_{queue}$ across 100 iterations. Figure 5b shows that the range of mean correlation coefficient (with $95 \%$ confidence) varies from 0.1 to 0.35, suggesting that mobility restrictions are indeed higher when the number of hospitalization rises.

**Fig. 6:**
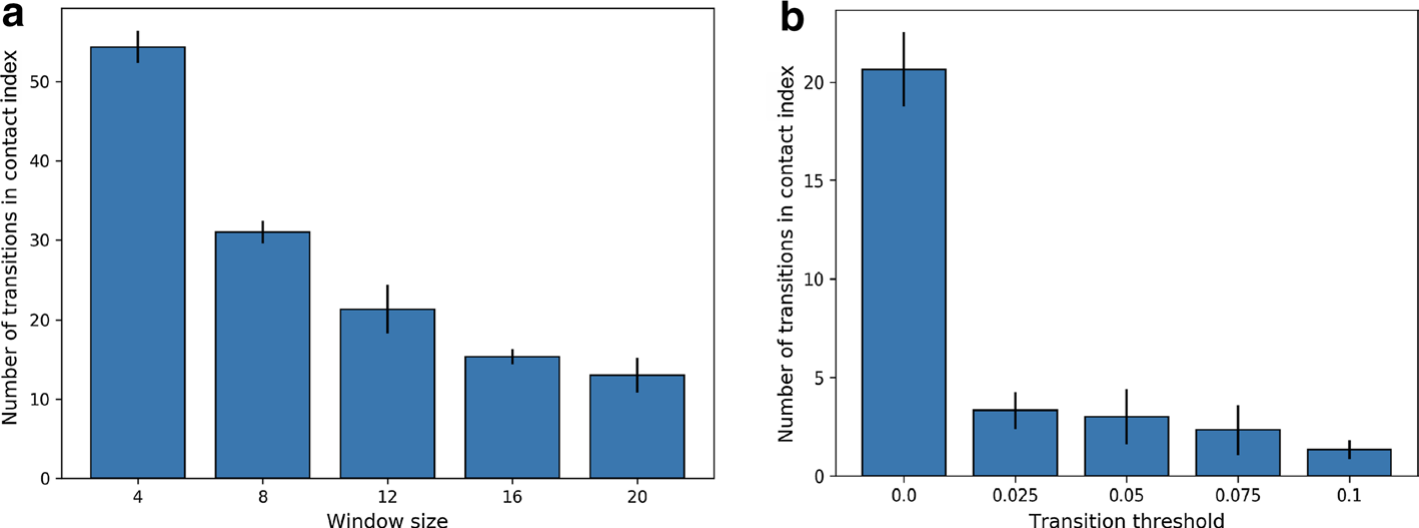
Cost and criteria for lockdown duration: variation in contact index for the increase in the **a** lockdown transition window $\tau$ and **b** threshold $\alpha$

#### Cost and criteria for lockdown duration

We discuss in “Overhead of lockdown” section that the duration of lockdown can be controlled by regulating the lockdown transition window $\tau$ and threshold $\alpha$. In Fig. 6a we show how the cost of lockdown (measured in terms of the number of transitions) in contact indices (*k*) decrease with $\tau = \kappa \times W + \frac{W}{2}$ hours, where $\kappa = 2, 4, 6, 8, 10$. Figure 6b shows that the decrease in $\alpha$ has a similar effect on transitions in *k*, increasing the overall cost of enforcing lockdown.

**Fig. 7:**
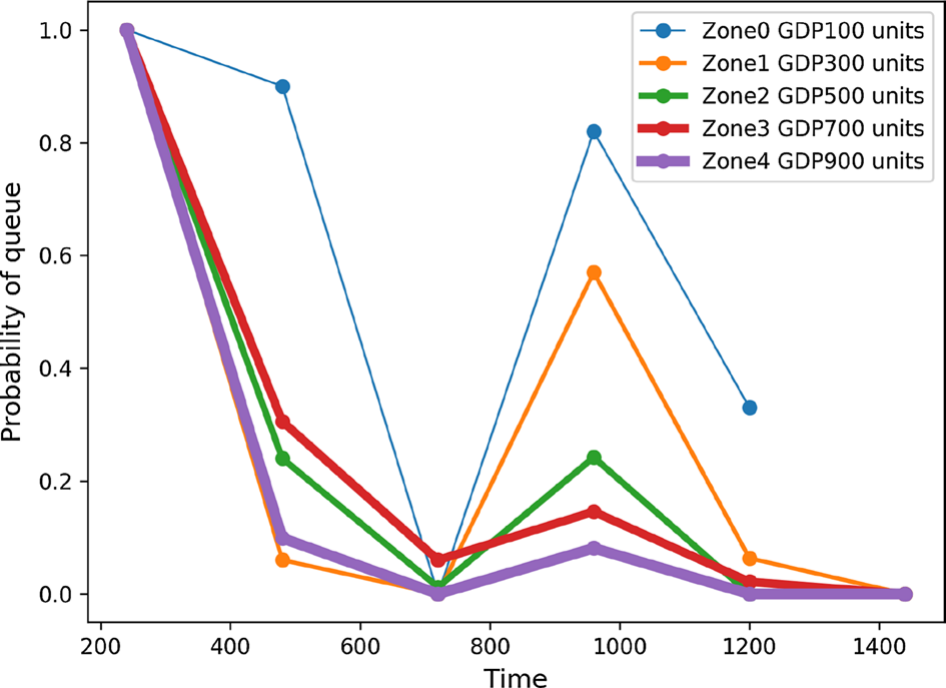
Probability of queues for identical zones, only varying in healthcare resource budget (measured in terms of GDP)

## Conclusions

We present an approach for dynamic time-varying lockdown strategy based on the healthcare budget and epidemic spread of a geographical region. This approach models each zone as an agent that applies reinforcement learning (RL) to periodically select a lockdown level that maximizes mobility, while constraining the number of hospitalizations to its healthcare resource budget. Through extensive simulation experiments on the real demographic and epidemiological data from the 5 boroughs of New York City, we demonstrate the efficacy of the approach. Each borough not only adapts to changing infection numbers by regulating its lockdown level, but also efficiently manages the overall cost associated with the time-varying dynamic lockdown strategy.

We are currently exploring how the RL model can employ epidemic model parameters, apart from mobility, that can realistically model infection spread via social contact. Second, here we assumed GDP as a measure of the healthcare resource available to a zone. To understand, how GDP affects the probability of hospital queue, we carry out a preliminary analysis where we create 5 zones that are identical in all respect, except have GDP $100, 200, \cdots , 500$ units, respectively. Figure 7 shows that regions with high GDP have a lower overall probability of queue. Taking a cue from this result, we shall devise collaborative strategies where neighboring zones with disparate healthcare budgets can pool their resources to avoid patient waiting times. This will require us to include additional considerations such as the distance between two zones as well as the quality of healthcare facility available to each zone based on some standard zone-level health index measures. Finally, we considered healthcare budget of a zone exclusively with respect to COVID-19 patients. This assumption may not always hold over long periods of time, making it imperative to include the effect of patients with other conditions into the RL model who may compete for the shared hospital resources.

## Data Availability

All relevant data (epidemiological and demographic data related to the boroughs of New York City) as well as the Python scripts are made available at https://github.com/satunr/COVID-19/tree/master/Dynamic_Lockdown

## Abbreviations

NYC: New York City
RL: Reinforcement learning
GDP: Gross domestic product
SEIRD: Susceptible exposed infected recovered dead
ODE: Ordinary differential equations
KL: Kullback–Leibler
